# Determination of the antimutagenicity of an aqueous extract of *Rhizophora mangle* L. (*Rhizophoraceae*), using *in vivo* and *in vitro* test systems

**DOI:** 10.1590/S1415-47572009005000106

**Published:** 2010-03-01

**Authors:** Maressa Malini, Maria Aparecida Marin-Morales, Mário Sérgio Mantovani, Claudia Masrouah Jamal, Natália Nati, Tatiane da Silva Passos, Silvia Tamie Matsumoto

**Affiliations:** 1 Departamento de Ciências Biológicas, Universidade Federal do Espírito Santo, Vitória, ESBrazil; 2 Departamento de Biologia, Universidade Estadual Paulista Júlio de Mesquita Filho', Rio Claro, SPBrazil; 3 Departamento de Genética, Universidade Estadual de Londrina, Londrina, PRBrazil; 4 Departamento de Ciências Farmacêuticas, Universidade Federal do Espírito Santo, Vitória, ESBrazil

**Keywords:** *Rhizophora mangle*, antimutagenicity, *Allium cepa*, CHO-K1

## Abstract

An aqueous extract of *Rhizophora mangle* L. bark is used as raw material in pottery making in the State of Espirito Santo, Brazil. This extract presents large quantities of tannins, compounds possessing antioxidant properties. Tannin antioxidant activity, as a plant chemical defense mechanism in the process of stabilizing free radicals, has been an incentive to studies on anti-mutagenicity. The present work aimed to evaluate possible antimutagenic activity of a *R. mangle* aqueous extract, using the *Allium cepa* test-system and micronuclear (MN) assay with blockage of cytokinesis in Chinese hamster ovary cells (CHO-K1). The *Allium cepa* test-system indicated antimutagenic activity against the damage induced by the mutagenic agent methyl methanesulfonate. A reduction in both MN cell frequency and chromosome breaks occurred in both the pre and post-treatment protocols. The MN testing of CHO-K1 cells revealed anti-mutagenic activity of the *R. mangle* extract against methyl methanesulfonate and doxorubicin in pre, simultaneous and post-treatment protocols. These results suggest the presence of phyto-constituents in the extract presenting demutagenic and bio-antimutagenic activities. Since the chemical constitution of *Rhizophora mangle* species presents elevated tannin content, it is highly probable that these compounds are the antimutagenic promoters themselves.

*Rhizophora mangle* L. (Rhizophoraceae) is a native mangrove species, with large quantities of tannins, mainly concentrated in the bark. According to Sanchez *et al.* (1998), the chemical constitution of an aqueous extract of *R. mangle* bark presents 54% in tannin content. On manufacturing the traditional clay cooking pots of Espírito Santo, and as part of the process followed by the Goiabeiras pot-makers association of Vitória, ES, Brazil, a *R. mangle* L. extract is used to cure the pots, thereby bestowing impermeability. In traditional medicine, the extract is also used for treating wounds, bacteriological inflammation and fungal diseases (Roig, 1974).

Ethnopharmacological reports are considered part of pre-triage strategy when searching for bioactive compounds in plants, since it is more likely that such compounds will be identified in plants already in use in traditional medicine than in randomly selected plants (Houghton and Raman, 1998). However, the realization of biological assays, *in vitro* and *in vivo*, is required to scientifically validate plants indicated by popular use.

*In vitro* tests are valuable in the study of medicinal plants, due to their greater reproducibility, shorter experimental periods and because they require smaller quantities of the target compounds. However, certain pharmacological actions also require the realization of *in vivo* tests, since these assays permit the analysis of other factors that influence the action of the active principal, such as administration route, absorption, metabolization and excretion (Calixto, 2001).

The mutagenic and antimutagenic potential of phytoconstituents present in plant and fungus extracts have been widely evaluated by means of *in vivo* assays involving *Allium cepa* and *in vitro* assays using Chinese hamster ovary (CHO-K1) cells.

Short duration tests involving the CHO-K1 system are indicated, due to the advantages offere, because the facility in standardizing experimental conditions (temperature, pH, culture medium composition and population density), the, metabolic and behavioral uniformity of the material, the possibility of undertaking cell treatment in several cell cycle phases, quickness, economy, adequate reproducibility, and chromosome and DNA organization equivalent to *in vivo* cells (Rodrigues and Monteleone-Neto, 1991). Assays using the genus *Allium* have been carried out since 1930, although their *modus operandi* was only standardized in 1985 by Fiskejö. The most frequently used species is *Allium cepa*, since the duration of its cell cycle is well-known, root growth is fast, cell division occurs in a large number of cells, it displays high tolerance to diverse culture conditions, it reacts to many known mutagenic agents, and its possesses only a few small-sized chromosomes (Evseeva and Khramova, 2002; Fiskejö, 1985; Matsumoto and Marin-Morales, 2004; Matsumoto *et al.*, 2006). The aim was to evaluate the cytotoxic and antimutagenic potential of an aqueous extract of *Rhizophora mangle* L. bark, used in the making of clay pots and in traditional medicine, by *Allium cepa* and cell testing, besides Chinese hamster ovary (CHO-K1) cell culture.

To prepare the aqueous extract of *Rhizophora mangle* L. bark, 300 g of plant bark were added to 900 mL of ultrapure water, where the bark remained immersed for 72 h. After this period, the solution was filtered, producing a crude extract of *R. mangle* bark at a concentration of 28 g/L. The extract obtained was submitted to pharmacognostic triage (Farmacopéia Brasileira, 2001). Tests were performed to identify flavanoids, terpenes, naphthoquinones, coumarin, saponins, anthracene heterosides, tannins and alkaloids. Determination of tannin dosage was performed using methods described in the Farmacopéia Brasileira *(2001*), with certain adaptation. To prepare the mother solution, 0.75 g of lyophilized *Rhizophora mangle* extract was dissolved in 150 mL of ultrapure water. The solution was then boiled, and maintained in a water bath at 80-90 °C for 30 min. After cooling and decantation, the sediment was filtered out. Total polyphenols (A1): 5 mL of the mother solution was transferred to a 25 mL volumetric balloon, whereupon the volume was completed with ultrapure water. In a 50 mL balloon, a 5 mL aliquot of this prepared solution was added to 2 mL of phosphotungstic acid. The balloon volume was completed with a sodium carbonate solution. Polyphenols not absorbed on skin powder (A2): 0.2 g of skin powder were added to 20 mL of the mother solution and stirred for 60 min. The resulting solution was filtered, and 5 mL diluted to 25 mL by adding ultrapure water. In a 50 mL volumetric balloon, 5 mL of the prepared solution was added to 2 mL of phosphotungstic acid. The balloon volume was completed with a sodium carbonate solution. Absorbance of solutions A1 and A2 was measured at 691 nm, 3 min after adding the last reagent, and using water as white-standard. The reference solution (A3) consisted of 50 mg of pyrogallol dissolved in 100 mL ultrapure water. In a 100 mL volumetric balloon, 5 mL of this solution were diluted to 100 mL using ultrapure water. In a 50 mL volumetric balloon, 5 mL of the latter was added to 2 mL of phosphotungstic acid. The balloon volume was completed with a sodium carbonate solution. The absorbance of solution A3 was measured at 691 nm, 3 min after the addition of the final reagent and within 15 min of dissolving the pyrogallol, with water as white standard. The tannin content was calculated by the equation



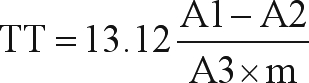


where TT = total tannins, A1 = mean absorbance of total polyphenols, A2 = mean absorbance of polyphenols not absorbed on skin powder, A3 = mean absorbance of the reference compound and m = mass of the lyophilized extract, in g.

The assay was done in triplicate and statistical analysis was performed by ANOVA (p < 0.05).

To determine the dilution of the extract for use in the antimutagenicity assays, preliminary toxicity tests were realized on *A. cepa* seeds, using various concentrations (28.0 g/L, 14.0 g/L, 7.0 g/L, 3.5 g/L and 1.75 g/L), to determine the concentration that would best respond to the antimutagenicity assays.

For each extract concentration tested, 25 seeds were left to germinate until root-length reached approximately 1 cm. Negative control was carried out with ultrapure water. Macroscopic parameters (root size and morphology) and the mitotic index were taken into consideration for evaluation of extract-toxicity. The roots were fixed and stained for cytological analysis by the Feulgen method (Mello and Vidal, 1978).

To evaluate the anti-mutagenic potential of *R. mangle* aqueous extract, DNA molecular damage was induced in meristematic cells of *Allium cepa* and CHO-K1 culture cells by methyl methanesulfonate (MMS) and doxorubicin (DXR). MMS is a mono-functional alkylating agent, with the capacity to generate methylating and ethylating species that interact with macromolecules, such as DNA. The MMS concentration used in CHO-K1 cell treatment was 4 x 10^-4^ M. DXR is a topoisomerase II inhibitor, which leads to the formation of simple and double breaks in the DNA chain and, consequently, to cell-death. It is believed that the disruption of DNA is also mediated by the generation of radicals favored by drug-free chemical structures (Chabner, 1996; Tokudome *et al.*, 2000). The DXR concentration used for CHO-K1 cell culture was 0.75 μg/mL of the culture medium.

Investigation of the antimutagenic activity of the *R. mangle* bark aqueous extract was carried out by pre-, simultaneous and post-treatment protocols, in the *Allium cepa* test-system and the CHO-K1 cell micronuclear assay, with cytokinesis blockage.

Seeds of *A. cepa* were germinated on a plate containing ultrapure water, until the roots had reached a length of approximately 1 cm. Next, the seeds were submitted to on of the following treatments: pretreatment, in which the seeds were transferred to a plate containing *R. mangle* bark aqueous extract at 1.75 g/L for 24 h, followed by treatment with mutagenic agent MMS (4 x 10^-4^ M) for a further 24 h; simultaneous treatment, in which the seeds were transferred to a plate containing *R. mangle* bark aqueous extract and mutagenic agent MMS in the proportion of 1:1 (1 extract:1 MMS, v:v) for 48 h; posttreatment, in which the seeds were transferred to a plate containing mutagenic agent MMS for 24 h, followed by treatment with *R. mangle* bark aqueous extract for 24 h. All the assays were performed in triplicate. Finally, all the meristems were fixed in Carnoy's solution (3:1) for 24 h. Cytological analysis was realized according to the Feulgen method; the roots were submitted to acid hydrolysis in 1 N HCl at 60 °C for 8 min, followed by washing in distilled water. Staining was realized with the periodic acid-Schiff reaction for 2 h in the dark. Root cells were then gently crushed and smeared onto the center of clean slides. 5000 cells per treatment were analyzed.

CHO-K1 cells, obtained from the Londrina State University (Universidade Estadual de Londrina, UEL) in October 2006, were used to perform the antimutagenic assay. Culture flasks (25 cm^2^) were used to seed monolayer cultures in D-MEM/HAM F12 medium, supplemented with 10% bovine fetal serum and 0.1% antibiotic-antimitotic solution. The flasks were maintained in a BOD incubator at 37 °C. Under these conditions, the cell cycle is 12 h. The different treatments were realized with *R. mangle* bark aqueous extract at a concentration of 1.75 g/L, after stabilization of CHO-K1 cell cultures for 48 h. Negative control was realized with PBS (pH 7.4) and positive controls with antineoplastic agent DXR (0.75 μg/mL of culture medium) and mutagenic agent MMS (4 x 10^-4^ M). The assays were realized in triplicate. For pretreatment the CHO-K1 cells were washed twice in PBS (pH 7.4), and cultivated in culture medium without serum supplementation with 50 μL of *R. mangle* extract for 3 h. Next, the cells were washed with PBS and submitted to treatment with either of the two positive controls for 3 h. For simultaneous treatment the CHO-K1 cells were washed twice in PBS (pH 7.4) and cultivated in culture medium with 50 μL of *R. mangle* extract and 50 μL of either of the two positive controls, where they remained for 3 h. In post-treatment the CHO-K1 cells were washed twice in PBS (pH 7.4) and cultivated in culture medium with either of the two positive controls for 3 h. Next, the cells were washed twice with PBS and 50 μL of *R. mangle* extract was added and where they remained for a further period of 3 h. At the end of each treatment, the mediums were discarded and the cells were washed twice with PBS. Culture medium and 50 μL of cytochalasin B were added. The CHO-K1 cells remained in cytochalasin for 18 h to induce the formation of binucleated cells. Following the cytochalasin B treatment, the CHO-K1 were again washed in PBS and detached with 0.0025% trypsin-EDTA. After trypsin inactivation with culture medium, one drop of formol was added and the sample was homogenized and centrifuged at 1250 rpm for 5 min. The supernatant was discarded and 1.5 mL of 1% sodium citrate were added. The sample was further homogenized and centrifuged at 1250 rpm for 5 min. The resulting pellet was resuspended in 5 mL of fixer (3 methanol:1 acetic acid, v:v), followed by centrifugation at 1250 rpm for 5 min. The fixer was removed until cell dilution for slide mounting was achieved, followed by staining with 5% Giemsa. 1000 cells per treatment were analyzed. Antimutagenic activity in the *Allium cepa* test-system and CHO-K1 cell culture were evaluated by analysis of the percentage reduction of DNA damage in each of the treatments with the aqueous extract of *R. mangle* bark, calculated according to the formula:



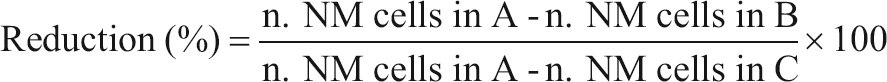


in which n = number of, A: DNA = damage-inducing agent, B = associated treatment and C = negative control.

Statistical analysis of the mitotic index was realized by the Kruskal-Wallis test. Statistical analysis of the number of micronucleated cells was realized by the Chi-square test. A p value of < 0.05 was considered significant.

*Rhizophora mangle* L. is a species that is widely encountered in Brazilian mangroves and has expressive importance in the economy of the State of Espirito Santo, Brazil, since the extract derived from its bark is used in the fabrication of traditional clay cooking pots. *R. mangle* bark is a rich red color, which gave origin to the popular name of mangue-vermelho (roughly translated: red mangrove). This coloration is derived from the presence of polyphenolic compounds, denominated tannins. These compounds are characterized by a reductive chemical structure that yields the capacity for free radical sequestration (Silva *et al.*, 2003).

Pharmacognostic triage detected the presence of flavonoids (5%), triterpenos (0.5%), anthracene heterosideos (traces) and hydrolyzable and condensed tannins (49.8%) The tannin dosage of the aqueous extract of *R. mangle* bark was 49.8% tannins (hydrolyzable and condensed), data that is in agreement with that obtained by Silva *et al* (Sanchez, 1998).

Evaluation of toxicity in *Allium cepa* revealed a decreasing dose-dependent relation between root growth and extract concentration, indicating that the toxicity of the extracts is proportional to an increase in the concentration of the same (Figure 1). This parameter indicated that extracts at concentrations of 1.75 g/L, 3.5 g/L and 7.0 g/L were adequate for use in biological experiments, since the roots originating from seeds germinated at these concentrations presented growth close to that of the negative control. For cytotoxicity analysis, the mitotic index of *A. cepa* roots was evaluated (Table 1). Observation verified that the number of cells presenting cell division was greater for the treatment with the lowest concentration, since at this concentration, greater root development occurred resulting from mitotic cell division. Following analysis of the two parameters evaluated, toxicity and cytotoxicity, the concentration of 1.75 g/L was determined as the fraction that should be used in the development of the mutagenicity and antimutagenicity studies.

Evaluation of possible antimutagenic activity of the *R. mangle* bark aqueous extract was realized by *in vivo* and *in vitro* assays, using the *A. cepa* test-system and CHO-K1 cell culture, respectively. In both the *A. cepa* test-system and CHO-K1 cell culture, *R. mangle* extract presented antimutagenic activity and absence of mutagenicity.

The antimutagenic effect was evaluated by pre-, simultaneous and post-treatment protocols to determine whether *R. mangle* extract acted as a demutagenic agent, *i.e.*, that it inactivated mutagenic agents, chemically or enzymatically, prior to their interaction with genetic material; and/or as a bio-antimutagenic agent, assisting in the capture of free radicals and potentiating DNA repair mechanisms (Kada *et al.*, 1982).

The *A. cepa* test-system indicated antimutagenic activity in pre and post-treatments with *R. mangle* extract at a concentration of 1.75 g/L, in relation to damage induced by the MMS mutagenic agent. The meristematic cells of *A. cepa* submitted to pretreatment with *R. mangle* extract presented a 95.1% reduction in micronuclear cell frequency and chromosome breaks induced by MMS. Under post-treatment conditions, there was an 85.0% reduction in DNA damage. On the contrary, no reduction in the rate of mutagenicity was observed during simultaneous treatment (Table 2).

The MN test in CHO-K1 cells detected antimutagenic activity for *R. mangle* extract (1.75 g/L) in all the treatments evaluated against the damage induced by the mutagenic agent MMS and the antineoplastic agent DXR. The reduction in the damage induced by MMS was lower in the pre and posttreatment protocols when compared to DXR; however, in the simultaneous treatment protocol, no difference in the rate of reduction was observed between the two agents (Table 2).

The hypothesis regarding the bio-antimutagenic and demutagenic activity of the *R. mangle* extract in the *A. cepa* test-system and CHO-K1 cell culture assays was supported by the presence of phytoconstituents in the extract that presented protective activity. Since the pharmacognostic triage and the dosage of the *R. mangle* bark aqueous extract determined that its chemical constitution presented a high content of tannins, it can be inferred that these polyphenols are the phytoconstituents that promoted the protective effect on the DNA molecules. The high antioxidant potential, which is characteristic of tannins, facilitates their acting through antimutagenic pathways, thereby capturing free radicals and contributing to the maintenance of cell stability (Picada *et al.*, 2003).

The results with *R. mangle* in *in vivo* and *in vitro* treatments revealed pronounced desmutagenic and bio-antimutagenic activity. The mechanism of demutagenic activity of *R. mangle* bark aqueous extract is also suggested by the interaction of tannin with metallic ions (Santos and Mello, 2003), thereby impeding that these ions participate in the reaction that transforms hydrogen peroxide into water, consequently inhibiting the formation of free radicals OH^-^ and hindering an attack on DNA molecules.

A possible mechanism of the bio-mutagenic action of the extract *R. mangle* is its actuation in the DNA repair system, that was implicated because the inhibition of the DNA topoisomerase II by the antineoplasic agent (Chabner, 1996; Tokudome *et al.*, 2000). In this way, the fixation of the mutations produced by the reactive oxygen species because the inefficiency of the topoisomerase II activity is decreased.

As the structure of chemical mutagens which favor the generation of free radicals result in increased oxidative stress, the primary desmutagenic action is to help intercept these radicals or even increase the production of antioxidant proteins to facilitate bio-antimutagenic action of tannins, thus reducing the amount of damage to be repaired.

During the simultaneous treatment, antimutagenic activity was not observed in the *A. cepa* test-system; however in the CHO-K1 cell culture test, the antimutagenic activity of the extract was observed for both DNA damage inductors (MMS and DXR). According to Rodrigues and Monteleone-Neto (1991), *in vitro* studies are the more advantageous by facilitating high-precision evaluation of the action of compounds at the cell level, because the experimental conditions are controlled. According to Calixto (2001), the effects observed in *in vitro* tests are frequently not in *in vivo*, due to difficulties in reproducibility in the latter.

Analysis of the present results suggests that aqueous extract of *Rhizophora mangle* bark used in the fabrication of domestic utensils by the pot-makers association of the city of Vitória, ES, Brazil, does not present mutagenic activity, rather it acted as a demutagenic agent in the *Allium cepa* test-system and CHO-K1 cells and as a bio-antimutagenic agent in CHO-K1 cell cultures.

**Figure 1:**
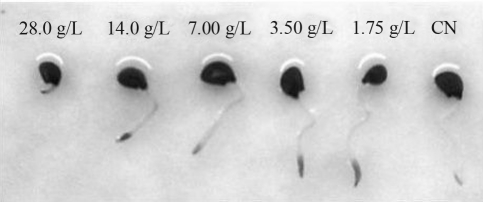
Growth of the roots of A. cepa seeds submitted to germination in different dilutions of R. mangle bark aqueous extract.

## Figures and Tables

**Table 1:** Mean values of the frequency of the mitotic indices of the meristems of *A. cepa* submitted to treatment with different concentrations of *R. mangle* bark aqueous extract.

Samples	Mitotic index (x)	Root length (mm)
Negative control	0.029	12.50
Extract 28.0 g/L	0.000	2.0
Extract 14.0 g/L	0.003	7.5
Extract 7.00 g/L	0.023	9.0
Extract 3.50 g/L	0.036	10.0
Extract 1.75 g/L	0.041*	17.0

*Statistically significant difference by the Kruskal-Wallis test for the mean root length, in relation to extract concentration 28.0 g/L.

**Table 2:** Frequency of cells with micronuclei and percentage reduction of chromosome damage in cells of *A. cepa* and CHO-K1 submitted to treatment with *R. mangle* bark aqueous extract

	Treatments	Frequency of cells with MN (1000 cells/experiment)					
		Experiments			X ± SD		% (chromosome damage)
		I	II	III			
*Allium cepa*	Negative control	0.0	0.0	1.0	0.33 ± 0.00		-
	MMS	7.0	8.0	5.0	6.67 ± 1.53*		-
	Extract 1.75 g/L	1.0	0.0	0.0	0.33 ± 0.00		-
	Pre MMS	0.0	0.0	1.0	0.33 ± 0.58**		95.1
	Simultaneous MMS	12	19	17	16.0 ± 3.61***		0.00
	Post MMS	1.0	1.0	1.0	1.00 ± 0.00**		85.0

CHO-K1	Negative control	1.0	1.0	1.0	1.00 ± 0.00		-
	MMS	27	28	47	34.0 ± 11.3*		-
	DXR	42	25	36	34.3 ± 8.62*		-
	Extract 1.75 g/L	0.0	1.0	2.0	1.00 ± 0.82		-
	Pre MMS	25	6.0	6.0	12.3 ± 11.0**		65.8
	Pre DXR	5.0	0.0	4.0	3.00 ± 2.65***		94.8
	Simultaneous MMS	8.0	12	7.0	9.00 ± 2.16**		76.0
	Simultaneous DXR	10	13	4.0	9.00 ± 3.37***		76.0
	Post MMS	19	21	25	21.6 ± 2.49**		38.1
	Post DXR	16	16	15	15.6 ± 0.47***		56.1

MMS, methyl methanesulfonate; DXR, doxorubicin; X, mean; SD, Standard deviation. *Statistical difference from negative control (p ≤ 0.05). **Statistical difference from MMS (p ≤ 0.05). ***Statistical difference from DXR (p ≤ 0.05).
